# A Promise in the Treatment of Endometriosis: An Observational Cohort Study on Ovarian Endometrioma Reduction by N-Acetylcysteine

**DOI:** 10.1155/2013/240702

**Published:** 2013-05-07

**Authors:** Maria Grazia Porpora, Roberto Brunelli, Graziella Costa, Ludovica Imperiale, Ewa K. Krasnowska, Thomas Lundeberg, Italo Nofroni, Maria Grazia Piccioni, Eugenia Pittaluga, Adele Ticino, Tiziana Parasassi

**Affiliations:** ^1^Dipartimento di Scienze Ginecologico-Ostetriche e Scienze Urologiche, Università di Roma Sapienza, Viale del Policlinico 155, 00161 Roma, Italy; ^2^Istituto di Farmacologia Traslazionale, CNR, Via del Fosso del Cavaliere 100, 00133 Roma, Italy; ^3^Foundation for Acupuncture and Alternative Biological Treatment Methods, Sabbatsbergs Hospital, Olivecronas väg 1, 113 24 Stockholm, Sweden; ^4^Dipartimento di Sanità Pubblica e Malattie Infettive, Università di Roma Sapienza, Viale del Policlinico 155, 00161 Roma, Italy

## Abstract

Urged by the unmet medical needs in endometriosis treatment, often with undesirable side effects, and encouraged by N-acetylcysteine (NAC) efficacy in an animal model of endometriosis and by the virtual absence of toxicity of this natural compound, we performed an observational cohort study on ovarian endometriosis. NAC treatment or no treatment was offered to 92 consecutive Italian women referred to our university hospital with ultrasound confirmed diagnosis of ovarian endometriosis and scheduled to undergo laparoscopy 3 months later. According to patients acceptance or refusal, NAC-treated and untreated groups finally comprised 73 and 72 endometriomas, respectively. After 3 months, within NAC-treated patients cyst mean diameter was slightly reduced (−1.5 mm) versus a significant increase (+6.6 mm) in untreated patients (*P* = 0.001). Particularly, during NAC treatment, more cysts reduced and fewer cysts increased their size. Our results are better than those reported after hormonal treatments. Twenty-four NAC-treated patients—versus 1 within controls—cancelled scheduled laparoscopy due to cysts decrease/disappearance and/or relevant pain reduction (21 cases) or pregnancy (1 case). Eight pregnancies occurred in NAC-treated patients and 6 in untreated patients. We can conclude that NAC actually represents a simple effective treatment for endometriosis, without side effects, and a suitable approach for women desiring a pregnancy.

## 1. Introduction

Endometriosis is one of the most frequent benign gynecological diseases, characterized by the implant and growth of viable endometrial tissue outside the uterine cavity, producing a general inflammatory response. The reported endometriosis prevalence in women of reproductive age ranges up to 10% [1]. In infertile women, the prevalence may rise to 50% [2]. Nevertheless, these numbers are likely to be underestimated.

The most common clinical symptoms include chronic lower abdominal and pelvic pain, dysmenorrhea, dyspareunia, abnormal uterine bleeding, and infertility.

Surgical removal of ectopic lesions represents the first-line intervention but is hampered by a relevant percentage of recurrences [3, 4]. In addition, a variety of medical hormonal therapies, all aimed to reduce the levels of circulating estrogens, are currently available [5]. However, these treatments are often unsatisfactory and cannot be used over long periods of time, due to the occurrence of severe adverse effects. Therefore, new and improved therapeutic solutions that can efficiently reduce lesions with limited side effects and no interference with the patient's fertility are definitely desirable.

We recently demonstrated that the well-known drug N-acetylcysteine (NAC), the acetylated form of the aminoacid cysteine naturally present in some substances like garlic [6], exerts a marked antiproliferative action *in vitro* on cancer cells of epithelial origin—the same origin of endometrial cells [7]. NAC action does not involve the induction of cell death nor is due to an unspecific toxic effect; rather, it is due to the establishment of a complex differentiation pathway, including the activation of several molecular mechanisms all converging into a proliferation-to-differentiation switch that implies a decreased cell proliferation and a decrease in cell locomotory behavior, particularly relevant in endometriosis. In addition, NAC also downregulates inflammatory protein activity and gene expression [8, 9]. Overall, NAC emerges as a thiol-containing compound active in the complex framework of redox signaling, with effects that are far beyond a generic antioxidant capability [10]. 

Translating *in vitro* findings into a murine model of endometriosis [11], we reported that NAC treatment induces a relevant reduction in endometriomas size, associated with decreased tissue inflammation and cell invasiveness. A similar effect of NAC in decreasing the production of hydrogen peroxide and in reducing cell proliferation was evidenced by using cellular and animal models of endometriosis and attributed to the regulation of the extracellular regulated kinase ERK1/2 [12]. 

Prompted by this background and with reference to both the unmet medical needs of endometriosis treatment and to the attractiveness of using NAC, virtually free of undesirable side effects and toxicity, we hereby report the successful treatment of ovarian endometriosis by using NAC.

## 2. Methods

Our observational cohort study was aimed at comparing the evolution of ovarian endometriomas in terms of mean diameter and volume, as evaluated by ultrasound, in NAC-treated and untreated control patients. The study protocol was approved by the Institutional Review Board of the University of Rome Sapienza (n.1451/11.09.08) and written informed consent was obtained from all patients. 

Patients were enrolled in the study according to the following inclusion criteria: (1) presence of an assigned ultrasound diagnosis of ovarian endometrioma; (2) no steroid treatment in the previous 2 months; (3) scheduled laparoscopy due to the presence of at least one of the following conditions: large ovarian endometrioma(s) with a mean diameter ≥30 mm, pain, and infertility. Diagnosis of ovarian endometriosis was issued with reference to an accurate medical history, pelvic examination (PV), and the combined use of transvaginal pelvic ultrasound (TVUS) and color Doppler evaluation [13, 14], performed in the early follicular phase of three consecutive menstrual cycles preceding enrollment [15]. Overall, this diagnostic procedure should grant almost a 100% diagnostic accuracy [16]. Ultrasound diagnosis of ovarian endometriomas was assigned when in the presence of the following established criteria: (1) a unilocular mass with ground glass echogenicity and a color score between 1 and 3; or (2) a unilocular mass with ground glass echogenicity with a papillary projection, a color score of 1 or 2 and no flow inside the papillary projection [13]. In some patients (6 in the treated group and 4 in the control group) with deep infiltrating lesions possibly involving the bowel or the bladder, MRI was also performed [17]. At enrollment, patients were allocated to the control or to the NAC-treated group according to their refusal or acceptance of the proposed treatment with NAC; routine laboratory tests were normal in all patients of the two groups. The observation period was of 3 months, corresponding to the mean duration of the waiting list for scheduled laparoscopy. At enrollment, routine laboratory tests were normal in all patients of the two groups.

At enrollment and at the end of the 3-month period of observation, endometriomas were evaluated by a trained physician with 20 years of experience in pelvic ultrasound (MGPi), blind to treatment but aware that the purpose of the examination was a precise monitoring of endometriomas dimension. All measurements were performed during the early follicular phase, that is, within the 8th day of the menstrual cycle, using a Siemens Sonoline Elegra scanner (Siemens, Issaquah, WA, USA), equipped with transabdominal (3.5 MHz) and transvaginal (5 MHz) probes; blood flow of the cysts was analyzed by the Color Doppler imaging. Pain symptoms (dysmenorrhea, dyspareunia, and chronic pelvic pain) were assessed by means of a 10-point visual analogue scale (VAS) both at enrollment and at the end of observation. Laparoscopy was performed under general anesthesia by the same surgeon with more than 20 years of experience (MGPo). Endometriosis was staged according to the revised classification of the American Society of Reproductive Medicine (rASRM) [18]. Histological examination was performed in all cases that ultimately underwent laparoscopy. 

NAC (Angenerico S.p.A., Ancona, Italy) was administered to patients *per os* for 3 months according to the following schedule: 600 mg three times a day, three consecutive days a week (Nactivia procedure, PMVENIRE, Stockholm, Sweden). The adoption of this procedure was based on the following considerations: (1) the daily total NAC dose of 1.8 g is virtually free of side effects and was already considered for other clinical indications [19]; (2) splitting the total dose in 3 is simple for patients and, with reference to the known NAC pharmacokinetics [20], grants a nearly constant plasma level of the drug; (3) the four-day medication-free interval provides a washout period useful to limit the reported decrease of NAC plasma level observed during prolonged treatments [20].

Immunohistochemical observation was performed on tissue samples obtained at laparoscopy from 2 NAC-treated and 2 untreated patients. Endometrioma tissue was processed using the method reported in [11]. Briefly, samples were fixed in 10% formalin, then paraffin-embedded, and 5 *μ*m-thick sections stained using haematoxylin-eosin. For antigen retrieval, sections were microwaved for 6 min in 0.1 M citrate buffer (pH 6.0) and endogenous peroxidase activity was blocked by 20 min incubation in 3% H_2_O_2_/methanol. Sections were rinsed in PBS/Triton X-100, immersed for 15 min in PBS-bovine serum albumin, and then incubated with the selected primary antibody. For immunodetection, Cox-2 rabbit polyclonal antibody was from NeoMarkers, Lab Vision Corporation (Thermo Fisher Scientific, Fremont, CA); E-cadherin and *β*-catenin were from BD Biosciences (Milan, Italy). DAKO Cytomation LSAB2 System-HRP (Dako Italia, Milan, Italy) was used to reveal antigens. After counterstaining with haematoxylin, sections were observed with a Zeiss Axioplan microscope. 

### 2.1. Statistical Methods

In a pilot study performed on a limited number of patients comprising a total of 32 ovarian endometriomas, we observed an average increase in the cyst volume of 5.9 mL in the NAC-treated group and of 30.1 mL in the untreated group, respectively, with a standard deviation SD = 69.4 in both groups. Given a first-type error *α* = 0.05 and a test power of 90%, the needed sample size in each group resulted in 67 cysts; evaluating an average of 7% of drop-out patients, we established a final requested sample size of 72 cysts in each group. The evaluation of the significance for average variations in cyst dimension within each group was performed using the Student's *t*-test for paired data. In order to evaluate whether these mean variations were different between the two compared groups, the Student's *t*-test was used for independent samples after a preliminary evaluation of the homogeneity of variance using the Levene's test. Given the heterogeneous variation in cyst dimensions that, within each of the two groups, could alternatively increase, decrease, or remain unchanged, we also compared NAC-treated and untreated cysts by using the Pearson's chi-square test. Significance was defined as *P* ≤ 0.05. Statistics were performed using SPSS software for Windows (release 13, 2004).

## 3. Results and Discussion

Between October 2008 and April 2011, 108 Italian women eligible for the study were consecutively enrolled; 7 patients in the NAC-treated group were not compliant to the scheduled therapy, and 9 patients in the control group dropped out. Therefore, a total of 92 patients, 47 in the NAC-treated group and 45 in the untreated group, were ultimately included in the study. Due to the contemporary presence of more than one cyst in several patients, we examined a total of 73 and 72 ovarian endometriomas in the NAC-treated and untreated group, respectively.

Patients in the two groups were comparable in terms of demographic characteristics, reproductive history, previous surgery for endometriosis, presence and severity of pain symptoms, and endometriomas size (Table 1). 

**Figure 1:**
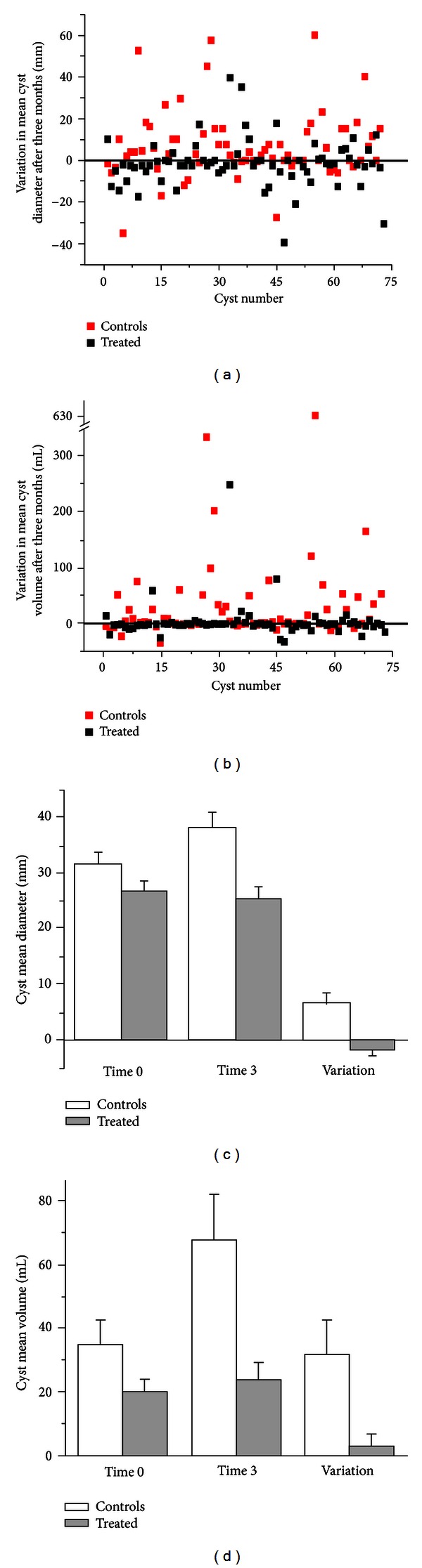
Variations in ovary cyst size after 3 months. Difference between the final and the initial mean diameters (a) and volumes (b) of all cysts in NAC-treated (black) and control (red) patients. Histogram of the averaged variations in mean diameter (c) and volume (d) in NAC-treated (gray) and control (white) patients. Statistical parameters are reported in detail in Table 2.

For both groups, raw values of the variation in cyst mean diameter (a) and volume (b) are shown in Figure 1; histograms of mean initial and final diameter (c) and volume (d), together with their respective variations, are also presented. Complete numeric values and statistical evaluations are detailed in Table 2. Notably, the significant increase in cysts size found in the control group was prevented by NAC.

As further outlined in Table 2, both in the NAC-treated and control group endometriomas alternatively decreased, increased, or remained unchanged; however, after NAC treatment more cysts underwent a size reduction and less cysts enlarged (*P* < 0.001). The number of reduced endometriomas also included disappeared cysts, 8 and 4 in NAC-treated and untreated patients, respectively; similarly, increased cysts included newly formed cysts, 4 and 7 in NAC-treated and untreated patients, respectively. 

The initial ovarian endometrioma dimension did not influence the response to treatment (*P* > 0.05). 

NAC was well tolerated by all patients and no adverse reactions were reported.

In the NAC-treated group, 24 patients cancelled the scheduled laparoscopy due to decreased (14 patients) or disappeared cysts (4 patients), pain reduction (21 patients), or pregnancy (1 patient). In the control group only 1 patient cancelled surgery. The rASRM stage, the disease score, and the pathology evaluation of all patients who ultimately underwent laparoscopy did not differ between the two groups (Table 3). 

The hypothesis tested in our study was to verify whether the evolution of ovarian endometriomas size could be positively influenced by NAC treatment during a relatively short interval of 3 months. Indeed, when compared to untreated controls, NAC treatment led to an increased number of cysts that either shrank or disappeared, as well as in a smaller number of enlarged and newly formed cysts. 

This effect is particularly remarkable because it favorably compares to the results reported in a randomized placebo controlled study performed on 100 patients treated for four months by using oral contraceptives (OC) [21]. Indeed, in this latter study, a reduction in cyst dimension was recorded not only in the OC-treated group but also in the placebo group. The overall reduction gain with OC (2.4 mL in volume and of 1.6 mm in average diameter) is far less impressive than that granted by NAC (28.9 mL in volume and of 8.2 mm in average diameter). A further comparison between OC and NAC treatments in terms of endometriomas stabilization, regression, or progression is made impossible by lack of disaggregated data in the OC study [21]. 

In NAC-treated and control patients we registered 6 and 8 pregnancies, respectively (Table 1); therefore, in striking contrast to all other available hormonal treatments of endometriosis (e.g., danazol, gonadotropin-releasing hormone analogues, progestins, and oral contraceptives) [22], NAC does not affect patient fertility and represents a suitable approach to treat endometriosis in women desiring pregnancy. Of the 8 pregnancies in the NAC-treated group, 1 occurred during treatment and 7 occurred within 2–12 months after completion of therapy (mean: 6 months). 

Although the pathogenesis of endometriosis is still debated [23], several evidences highlight that both the onset and progression of this disease are supported by a derangement of invasive, proliferative, adhesive, and locomotory properties of endometrial cells, along with an increased production of inflammatory molecules [24]. In this regard, our rationale for testing NAC effectiveness in the clinical management of endometriosis was granted by our previous evidences showing that NAC, through its action on thiol redox signaling [10], positively modulates several pathways relevant in the pathogenesis of endometriosis [7–9, 11]. Indeed, *in vitro*, NAC decreases abnormal cell proliferation, cell locomotory characteristics, expression/activity of some inflammation-related genes, and stimulates terminal cell differentiation, that is, a finite cell lifespan [7–9]. Consistently, in an animal model, NAC treatment results in a reduction in size of experimentally induced endometriomas through a direct action on the endometrial tissue that displays (1) a differentiated morphology; (2) a decrease in cell invasive behavior, evidenced by an increase in cell-cell junctions and a decreased expression of metalloproteinases; and (3) a relevant decrease in the cyclooxygenase-2 (COX-2) gene and protein expression [11].

**Figure 2:**
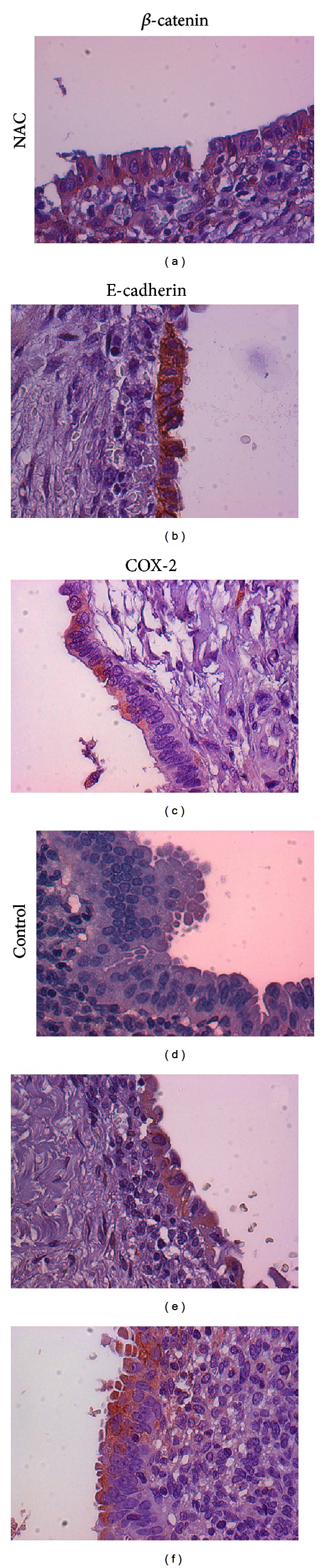
Immunohistochemical comparison of NAC-treated and untreated endometrioma tissue obtained at laparoscopy. Representative examples of NAC-treated (a, b, and c) and untreated (d, e, and f) tissue samples showing an increased labeling for proteins belonging to the cell-cell adhesion complexes, *β*-catenin (a) versus (d) and E-cadherin (b) versus (e) and a decrease in inflammatory COX-2 (c) versus (f). Original magnification 40x.

In agreement with these evidences, preliminary immunohistochemical examinations of cyst tissue obtained at laparoscopy anticipate that similar molecular mechanisms are at play also in the human and substantiate the present clinical findings. Indeed, in tissue from NAC-treated patients we observed (Figure 2) (1) a more differentiated morphology of the epithelial layer; (2) an increase in proteins of cell-cell junction complex such as E-cadherin and *β*-catenin, indicating a decrease in cell invasive behavior; and (3) a decrease in the inflammatory COX-2. This latter evidence suggests a possible explanation of the observed reduction in cysts dimensions. Indeed, a decrease in COX-2 ultimately reduces estrogens availability in the ectopic endometrium through a decreased production of prostaglandin E2—the most powerful stimulator of aromatase [25]. Of note, we foresee that NAC action on cell signaling and protein activity, with the overall effect of reverting a deranged proliferation to a physiological behavior, can help in preventing recurrences. However, both a complete elucidation of the mechanisms activated by NAC and its possible role in the prevention of recurrences deserve future wider studies.

Due to the bias represented by the absence of a placebo group, the observed variations in pain incidence and severity cannot be validated; however, while dysmenorrhea, dyspareunia, and chronic pelvic pain were similar in the two groups at enrollment, they were all significantly reduced in the NAC-treated group at the end of observation, with a decrease of 55% in dysmenorrhea (*P* = 0.001), of 50% in dyspareunia (*P* = 0.027) and of 59% in chronic pelvic pain (*P* = 0.015) (Table 4). These data definitely deserve a validation in placebo-controlled future studies; of note, pain amelioration represented the main reason for surgery cancellation in the NAC-treated group (21/47).

## 4. Conclusions

In conclusion, with reference to the unmet medical needs of endometriosis, our results clearly show that, by targeting various molecular and biochemical pathways involved in the initiation and maintenance of this disease, NAC effectively treats ovarian endometriosis. In terms of reduction in cysts size, our data are even more favorable than those granted by the currently adopted hormonal treatments, with the further advantages of fertility preservation and of the virtual absence of undesired side effects. The low cost of this natural drug can also be of interest for public health institutions.

## Figures and Tables

**Table 1:** Characteristics of patients.

	Controls (*n* = 45)	Treated (*n* = 47)	*P*
Age (years)	32.9 ± 6.5	32.5 ± 7.3	ns
Weight (kg)	57.8 ± 7.2	60.2 ± 13.3	ns
Height (cm)	164.3 ± 5.8	163.8 ± 6.8	ns
BMI	21.4 ± 2.5	22.5 ± 4.9	ns
Past hormonal therapy (*n*)	22	28	ns
Pregnancy desire (*n*)	14	15	ns
Parity (*n*)			
0	29	35	ns
1	11	10	ns
≥2	5	2	ns
Pregnancies post (*n*)^a^	6	8	ns
Infertility	10	13	ns
Previous surgery for endometriosis	17	12	ns
Deep infiltrating lesions	4	2	ns
Dysmenorrhea (1–10 VAS)	7.18 ± 2.58 (*n* = 42)	6.43 ± 3.39 (*n* = 38)	ns
Dyspareunia (1–10 VAS)	2.76 ± 3.27 (*n* = 20)	2.83 ± 3.19 (*n* = 23)	ns
Chronic pelvic pain (1–10 VAS)	2.07 ± 3.57 (*n* = 12)	2.47 ± 3.82 (*n* = 15)	ns
Patients with more than 1 cyst	21^b^	20^c^	ns

	(*n* = 72)	(*n* = 73)	

Cyst size			
Average diameter (mm)	31.6 ± 18.4	26.9 ± 14.9	ns
Average volume (mL)	35.1 ± 62.6	20.4 ± 31.3	ns

^a^Only 1 pregnancy in the treated group was reported during the 3 months. All other pregnancies occurred after the observation period and were reported by the patients. ^b^ *n* = 17: 2 cysts; *n* = 3: 3 cysts; *n* = 1: 5 cysts. ^c^ *n* = 15: 2 cysts; *n* = 4: 3 cysts; *n* = 1: 4 cysts.

**Table 2:** Endometriomas size at enrollment and at the end of observation.

	Controls (*n* = 72)	Treated (*n* = 73)	*P*
Average diameter (mm)			
Initial	31.6 ± 18.4	26.9 ± 14.9	ns
Final	38.3 ± 23.2	25.4 ± 17.8	<0.001
Difference	6.62 ± 16.14	−1.53 ± 11.43	0.001
Average volume (mL)			
Initial	35.1 ± 62.6	20.4 ± 31.3	ns
Final	67.3 ± 123.2	23.6 ± 48.6	0.006
Difference	32.1 ± 89.9	3.20 ± 32.7	0.012
Number of cysts			
Increased	42 (58%) (7 newly formed)	20 (27%) (4 newly formed)	<0.001*
Decreased	20 (28%) (4 disappeared)	45 (62%) (8 disappeared)	
Unchanged	10 (14%)	8 (11%)	

*At the Pearson's chi-square test.

**Table 3:** Patients disease stage and score at laparoscopy.

	Controls (*n* = 44)	Treated (*n* = 23)	*P*
rARMS score	39.6 ± 27.5	49.7 ± 32.6	ns
rARMS stage			
II	4 (9%)	2 (9%)	ns
III	28 (64%)	15 (65%)	ns
IV	12 (27%)	6 (26%)	ns

**Table 4:** Pain evaluation at enrollment and at the end of observation (1–10 VAS scale).

	Controls	Treated	*P*
Dysmenorrhea			
Initial	7.18 ± 2.58 (*n* = 42)	6.43 ± 3.39 (*n* = 38)	ns
Final	7.00 ± 2.79 (*n* = 41)	3.11 ± 3.33 (*n* = 24)	0.001
Dyspareunia			
Initial	2.76 ± 3.27 (*n* = 20)	2.83 ± 3.19 (*n* = 23)	ns
Final	2.78 ± 3.31 (*n* = 20)	1.38 ± 2.62 (*n* = 12)	0.027
Chronic pelvic pain			
Initial	2.07 ± 3.57 (*n* = 12)	2.47 ± 3.82 (*n* = 15)	ns
Final	1.87 ± 3.42 (*n* = 11)	0.77 ± 2.09 (*n* = 6)	0.015
